# Advanced Respiratory Motion Compensation for Coronary MR Angiography

**DOI:** 10.3390/s130606882

**Published:** 2013-05-24

**Authors:** Markus Henningsson, Rene M. Botnar

**Affiliations:** 1 Division of Imaging Sciences & Biomedical Engineering, the Rayne Institute, King's College London, 4th Floor, Lambeth Wing, St Thomas' Hospital, London, SE1 7EH, UK; E-Mail: rene.botnar@kcl.ac.uk; 2 Wellcome Trust and ESPRC Medical Engineering Center, King's College London, London, SE1 7EH, UK; 3 BHF Centre of Excellence, King's College London, London, SE1 7EH, UK; 4 NIHR Biomedical Research Centre, King's College London, London, SE1 7EH, UK

**Keywords:** magnetic resonance imaging, coronary magnetic resonance angiography, respiratory motion correction, respiratory navigation

## Abstract

Despite technical advances, respiratory motion remains a major impediment in a substantial amount of patients undergoing coronary magnetic resonance angiography (CMRA). Traditionally, respiratory motion compensation has been performed with a one-dimensional respiratory navigator positioned on the right hemi-diaphragm, using a motion model to estimate and correct for the bulk respiratory motion of the heart. Recent technical advancements has allowed for direct respiratory motion estimation of the heart, with improved motion compensation performance. Some of these new methods, particularly using image-based navigators or respiratory binning, allow for more advanced motion correction which enables CMRA data acquisition throughout most or all of the respiratory cycle, thereby significantly reducing scan time. This review describes the three components typically involved in most motion compensation strategies for CMRA, including respiratory motion estimation, gating and correction, and how these processes can be utilized to perform advanced respiratory motion compensation.

## 1. Introduction

**Figure 1.**
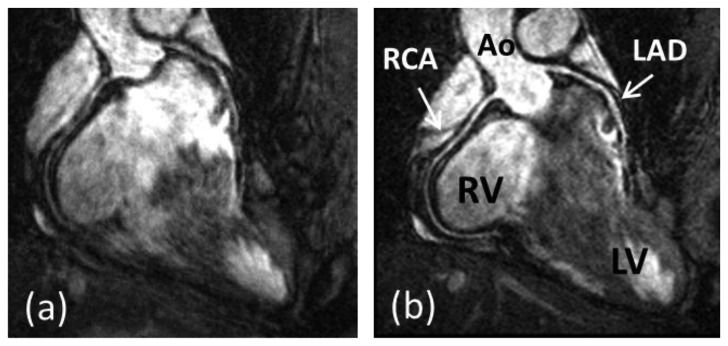
CMRA dataset without respiratory motion correction (**a**) and with respiratory motion correction (**b**). The 3D dataset is reformatted to visualize the right coronary artery (RCA) and left anterior descending artery (LAD). Ao = ascending aorta; RV = right ventricle; LV = left ventricle. Adapted from [11].

Coronary artery disease (CAD) is the leading cause of death in the Western world and developing countries [1]. The gold standard imaging modality for detection of CAD is X-ray angiography, which is invasive, exposes patients to potentially harmful radiation and requires iodinated contrast agents. Multi-Detector Computed Tomography (MDCT) has been shown to be a reliable technique for non-invasive detection of coronary artery stenosis [2]. However, MDCT, like X-ray, utilizes ionizing radiation. Furthermore, the diagnostic accuracy of MDCT is reduced in patients with heavily calcified plaques. In recent decades coronary magnetic resonance angiography (CMRA) has emerged as a non-invasive alternative for the diagnosis of CAD without exposing patients to potentially harmful radiation or nephrotoxic contrast agents. But despite advancements in CMRA hardware and software techniques, artifacts due to residual coronary artery motion remain an impediment to diagnostic CMRA in a small but significant amount of patients. Several studies have investigated respiration induced coronary motion along the foot–head (FH), left–right (LR), and anterior–posterior (AP) directions throughout the respiratory cycle [3–6]. Although the principal motion component is predominately in the FH direction and is often one order of magnitude larger than the CMRA spatial resolution, the displacement magnitude in the LR or AP direction has high subject specific variability and can contribute to motion artifacts in CMRA. Furthermore, rotation and non-rigid motion such as affine or even non-linear deformation may occur between the end-expiratory and end-inspiratory respiratory phase [7–10]. As a result, CMRA is particularly susceptible to respiratory motion artifacts due to the relatively slow data acquisition speed of MRI and the high spatial resolution required to visualize the coronary artery lumen. The main coronary arteries, which consist of the right coronary artery (RCA) and the left main (LM) artery which branches into the left anterior descending (LAD) artery and left circumflex (LCX) artery, have a diameter of approximately 3 mm in the proximal coronary segments which reduces to 1 mm in more distal segments. A respiratory motion corrupted CMRA dataset is shown in Figure 1(a), along with the same dataset after respiratory motion correction in Figure 1(b), reformatted to visualize the RCA and LAD.

For diagnostic purposes it is desirable to detect obstructive coronary stenosis of at least 50% reduction of the coronary lumen. Thus, CMRA spatial resolution is typically on the order of 0.7 to 1.3 mm. This extends the CMRA scan time beyond what can be comfortably acquired within a breath-hold. In comparison, due to superior acquisition speed, X-ray angiography and MDCT allows for data acquisition with sub-millimeter resolution within a single breath-hold, which effectively minimizes the problem or respiratory motion. Although respiratory motion compensation in the early days of CMRA involved breath-holding [12], image acquisition typically only employed two-dimensional (2D) encoding and therefore provided limited coverage of the coronary arteries [13–15]. As the coronary arteries are long and tortuous, three-dimensional (3D) coverage is important to visualize one or more coronary arteries in a single scan. Developments in MRI acceleration techniques including partial Fourier [16] and parallel imaging [17,18] have enabled 3D CMRA data acquisitions within a single breath-hold [19–21]. Further acceleration may be achieved with compressed sensing [22–24] although its efficacy in conjunction with breath-hold CMRA has yet to be established. Notwithstanding these technical developments, the relatively long breath-hold durations of approximately 20–25 s may be difficult to achieve in patients with CAD which is why free-breathing motion compensated CMRA approaches are typically favoured.

**Figure 2.**
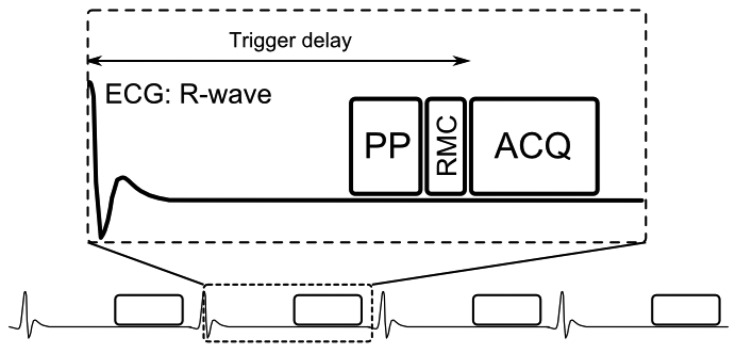
Schematics of ECG-triggered CMRA sequence. One k-space data segment is acquired (ACQ) following a time delay (trigger delay) after the R-wave to minimize cardiac motion artifacts. Typically the trigger delay is adjusted to coincide with the mid-diastolic rest period and the center of k-space. Preparation pulses (PP) can be used to improve visualization of the coronary arteries by suppressing signal from surrounding tissues, including epicardial fat and myocardium. Respiratory motion compensation (RMC), including motion estimation, gating and correction, is typically performed prior to ACQ, although gating and correction may be also being performed retrospectively.

A generic CMRA sequence is shown in Figure 2, where the segmented k-space data acquisition is synchronized with the ECG to minimize cardiac motion related artifacts [25]. Various magnetization preparation pulses such as T2prep [26] and fat suppression [27] are used to improve visualization of the coronary lumen, and respiratory motion compensation is employed to suppress respiratory motion artifacts. There are essentially three separate processes for respiratory motion compensation which can be used separately or in concert in CMRA to reduce respiratory motion artifacts: (i) motion estimation, (ii) gating and (iii) correction. The focus of this review is to describe these motion compensation processes. However, it is worth noting that there are alternative methods to minimize motion artifacts in MRI, for example by signal averaging or using motion insensitive *k*-space trajectories such as radial [28,29] or spiral [30] sampling.

## 2. Respiratory Motion Estimation

Most respiratory compensation strategies rely on some sort of motion estimation information as input to the gating or correction algorithms. There are a number of ways in which motion estimation can be achieved, from external monitoring devices to MRI based techniques, so called respiratory navigators.

### 2.1. External Devices

The most commonly used external respiratory monitoring device in cardiovascular MRI is the respiratory bellows [31,32], which measures pressure differences between the abdomen and a tightly wrapped belt to establish whether the respiratory cycle is in inspiration or expiration. Other techniques to perform respiratory motion estimation with external devices include measuring the electric impedance between ECG leads [33] or the more recently developed ultra wideband electromagnetic sensor [34]. The advantage of utilizing external devices for respiratory motion estimation is that the motion information may be obtained with a very high temporal resolution due to the low dimensionality of the measurements. Therefore, external devices can be useful in conjunction with simple respiratory gating such as the accept/reject algorithm which will be described in the following section. An additional advantage of external devices is that they are independent of the MRI acquisition and can be acquired in parallel with the CMRA, which is particularly important for steady-state MRI sequences. Nevertheless, for high-resolution imaging such as CMRA, external devices alone may be insufficient to adequately compensate for the respiratory motion. This is because they typically only provide relative information about respiration, which can be used to determine the respiratory phase (inspiration or expiration) but not any absolute motion information, such as displacement, rotation, *etc.* Therefore, its usefulness for the purpose of respiratory motion correction is very limited.

### 2.2. Respiratory Navigators

Respiratory navigators are integrated into the MRI pulse sequence and can be used to monitor the respiratory displacement and deformation of the heart and surrounding tissues and organs. Respiratory navigators are typically separate real-time image acquisitions, interleaved with the high resolution CMRA sequence, providing snap-shots of one or more motion dimensions of the respiratory state before or after each segmented *k*-space acquisition. There exists a multitude of variations and implementations, which will be discussed in more detail.

#### 2.2.1. Diaphragmatic 1D Navigators

The first respiratory navigator was proposed in the late 1980's by Ehman *et al.* [35] and consisted of a 1D signal which was acquired on the dome of the right hemi-diaphragm, measuring the FH motion of the lung-liver interface to correct for the respiratory motion of the liver. Since then, diaphragmatic 1D navigators (d1D NAV) have become increasingly popular as a way of estimating respiratory motion for a wide range of cardiovascular MRI applications due to its compatibility with a large number of cardiovascular imaging sequences, relative ease of use and sharpness of the lung-liver interface [36]. A further advantage of the d1D NAV is that the acquisition and processing time is short (on the order of 10–30 ms), making it suitable for prospective real-time motion compensation. Moreover, respiratory motion has been shown to be predominant in the foot-head direction which is orthogonal to the lung-liver interface [3] which may be accurately tracked with a d1D NAV approach.

**Figure 3.**
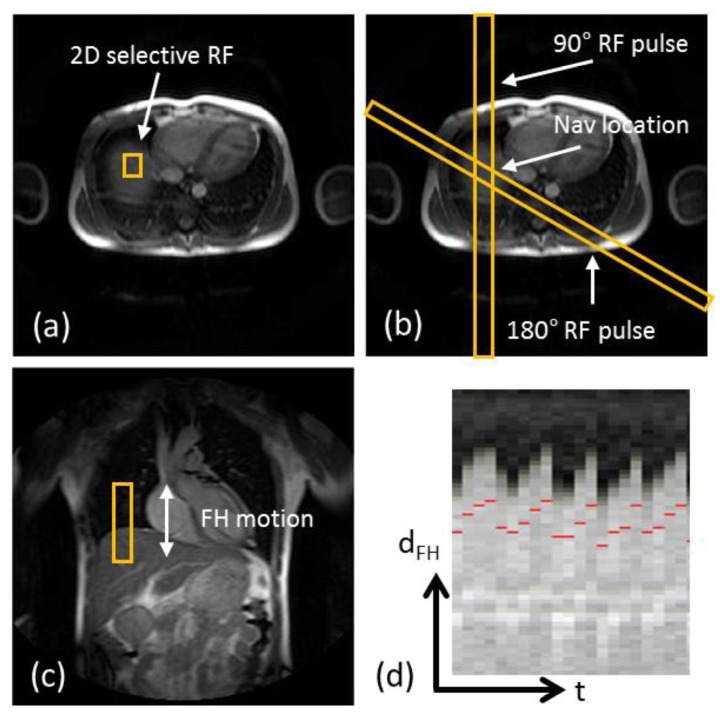
Scan planning of d1D NAV in axial plane using either a 2D selective RF “pencil beam” excitation (**a**) or a spin-echo approach with obliquely aligned 90° excitation and 180° refocusing pulses (**b**). The d1D NAV positioned in the coronal plane, on the dome of the right hemi-diaphragm with the readout in foot-head (FH) direction (**c**). The d1D NAV signal (**d**) clearly captures the displacement of the lung-liver interface along the FH direction (d_FH_) over time (t).

Implementations of the d1D NAV involves either a 2D radiofrequency (RF) excitation pulse with a spoiled gradient echo read-out in the orthogonal direction [37,38], a so called pencil beam navigator, or a spin-echo approach where the 90° RF excitation and 180° RF refocusing pulse are obliquely aligned and the d1D NAV volume is defined as the overlapping area [35,39]. Figure 3 shows images of scan planning using either of the previously mentioned techniques to define the d1D NAV volume, as well as the resulting d1D NAV signal over time, clearly capturing the respiratory motion of the lung-liver interface. Although the 1D navigator may be used to track the respiratory motion directly on the heart with similar motion compensation efficacy, studies have found the diaphragm to be the optimal location due to the reduced signal-to-noise of 1D navigators positioned on the heart [36,40] and avoiding the risk of signal saturation or interference between the overlapping navigator and CMRA acquisitions. However, problems associated with d1D navigators include hysteresis between the respiratory motion of the diaphragm and the heart between inspiration and expiration [41], as well as the indirect estimation of the respiratory motion of the heart which necessitates a motion model to translate the diaphragmatic respiratory motion to that of the heart. Although respiratory motion of the heart throughout the respiratory cycle can be approximated by 3D affine transformations [7], early studies using d1D navigators investigated the linear relationship between the respiratory motion of the diaphragm and that of the heart [3–5,42]. A large inter-subject variability was reported with regard to the linear relationship of the diaphragm-heart motion, as well as a substantial variability relating to the location on the heart where the motion comparison was made due to the non-rigid respiratory induced deformation of the heart [43]. Improved motion estimation has been demonstrated using subject-specific linear motion models adapted to certain anatomic regions on the heart [44]. Nevertheless, a linear model with a constant factor of 0.6 in FH direction has been established as a generally acceptable motion model between the diaphragm and the heart, and has been used in most clinical studies using free-breathing CMRA [45–49]. More recent studies using d1D navigators have been able to obtain patient specific models between the respiratory motion of the diaphragm and the 3D affine motion of the heart from calibration pre-scans [50,51]. However, such subject specific motion models are typically non-trivial to generate and further complicates the cardiac examination due to the requirement of calibration scans.

#### 2.2.2. Self-Navigation

A further limitation of the d1D NAV is the added scan complexity it requires, including scan planning and defining separate imaging parameters for the navigator acquisition, on top of the already complicated CMRA examination. These limitations have motivated a recent trend in respiratory motion compensation, so called self-navigation [52–55]. In this approach respiratory motion can be estimated directly from the CMRA data, provided that a few conditions are met. The most important requirement is that the central line of k-space is repeatedly measured, at least once per k-space segment, and ideally the readout should be aligned with the FH direction to maximize the sensitivity for respiratory motion. Self-navigation has been implemented to allow for respiratory motion estimation along the FH direction, however 3D self-navigation has been proposed as well [56]. An advantage of self-navigation is that respiratory-induced motion of the heart can be measured directly, which obviates the need for a diaphragmatic motion model. However, as self-navigation images are typically acquired as 1D projections of the FOV, static tissue such as the chest wall is also included in the navigator image and may reduce the motion estimation performance of the navigator. Although methods have been proposed to suppress signal from static tissue for 1D self-navigation [54,56], an extension of this method overcomes this problem by separating moving from static tissue in the navigator using spatial encoding, so called image-based navigation.

#### 2.2.3. Image-Based Navigation

In image-based navigation single 2D [57,58], orthogonal 2D [59], or 3D [60] real-time images are acquired in every heart-beat prior to CMRA acquisition. The main advantage of this approach is that the moving heart can be spatially isolated from surrounding static tissues, which allows for improved respiratory motion estimation. Furthermore, image-based motion compensation has intrinsically more degrees of freedom for motion correction, which is not limited just to translational motion in several directions but also includes rotation and non-rigid motion correction. A potential risk of using image-based navigation is that the navigator acquisition may saturate signal or cause artifacts in the subsequent and spatially overlapping CMRA acquisition. One solution to this problem is to use spectrally selective RF pulses for the navigator to image and estimate motion from the epicardial fat, while the CMRA acquisition relies on water signal from blood and tissues [61]. An alternative is to use a so called trailing navigator, where the navigator image acquisition is performed after the CMRA acquisition in the cardiac cycle. However, this approach is incompatible with prospective respiratory gating and correction. The feasibility of prospective image-based motion correction has recently been demonstrated [57,58], although compared to 1D navigation such as the diaphragmatic navigator or self-navigation the increased computational complexity of multi-dimensional image reconstruction, registration and correction makes real-time implementations more challenging. A solution to this problem could be to implement the navigator image post-processing on graphics processing units [62,63] as most of the steps can be parallelized. However, even more crucial for accurate motion estimation using image-based navigation is the close temporal proximity of the navigator with the CRMA sequence to ensure good correlation between the estimated motion at the time of the navigator acquisition and the actual motion during the CMRA acquisition. Spuentrup *et al.* found that a short time delay between the navigator acquisition and the imaging, *i.e.*, temporal proximity, was critical to minimize motion artifacts in high resolution MRI, whereas the navigator spatial resolution was of minor importance [64].

**Figure 4.**
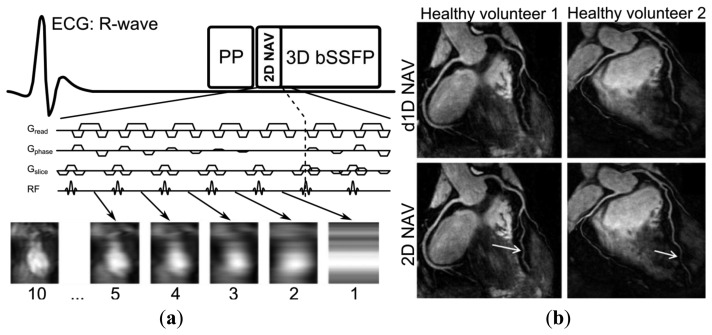
Sequence diagram of two-dimensional image-based self-navigation (2D NAV) using the startup echoes of a 3D balanced Steady-State Free Precession (bSSFP) sequence (**a**). The 2D NAV is generated by adding phase encoding gradients (G_phase_) to the startup echoes, using a high-low profile order. The number of startup echoes is proportional to the 2D NAV phase encoding resolution, however 10 startup echoes are commonly used which results in accurate respiratory motion estimation of the heart. Whole-heart CMRA from two healthy volunteers using either the conventional diaphragmatic 1D navigator (d1D NAV) with a tracking factor of 0.6 for motion estimation (top row) or 2D NAV (bottom row) with improved coronary vessel sharpness of the distal LAD (arrows) for the 2D NAV approach (**b**). Adapted from [65].

The main drawback of image-based navigation compared to self-navigation is that the navigator may not be as readily extracted from the image acquisition which means that additional scan planning is necessary to define the navigator image location. To combine the advantages of self-navigation with those of image-based navigation, an approach has been proposed whereby the navigator signal is acquired using the startup echoes [65] of a balanced steady-state free precession CMRA sequence [66]. In this approach, the navigator has nearly identical imaging parameters including location and orientation (self-navigation) while still generating a 2D image, by spatially encoding the startup echoes by adding phase encoding gradients (image-based navigation), as shown in Figure 4. Another image-based self-navigation approach divides the respiratory cycles into several states (so called bins), whereby undersampled radial CMRA data from several cardiac cycles but the same respiratory bin can be reconstructed and used to estimate respiratory motion between bins [67,68]. Similar approaches, using respiratory binning, have been implemented for Cartesian sampling as well. However, in these approaches the respiratory motion estimation is based on data acquisition performed in addition to the CMRA data [11,69]. All binning approaches require an initial motion estimation to define the respiratory bin for each cardiac cycle, typically with the d1D NAV, and thereafter the respiratory motion is estimated from the heart motion between respiratory bins.

## 3. Respiratory Gating Strategies

**Figure 5.**
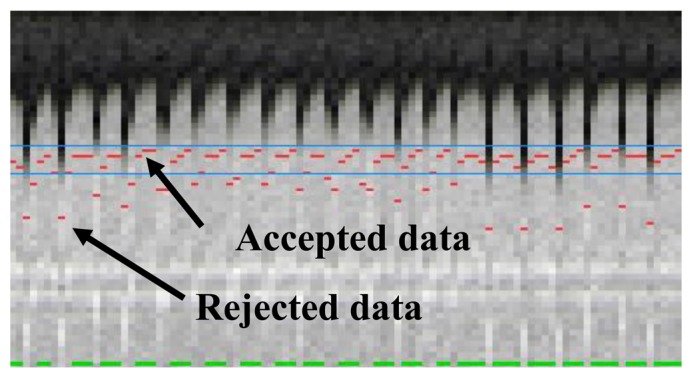
Respiratory 1D navigator signals throughout a scan. One column represents one navigator acquisition, and the red dot the measured respiratory position. Image data acquired at positions outside of the gating window, shown by the blue lines, are rejected and re-acquired in the following shot and only accepted once the corresponding navigator has been acquired within the gating window which is signified by the green lines at the bottom of each navigator image. Although this method effectively reduces the respiratory motion artifacts, it prolongs the scan, typically by a factor of 2 or more.

The most commonly used approach for respiratory gating is to limit image acquisition to the most quiescent respiratory phase, which usually is end expiration, and reject data acquired outside of the “respiratory gating window”. Figure 5 shows a time series of diaphragmatic 1D navigator signals during a scan, including the respiratory gating window (blue lines) within which acquired image data is accepted. This method, called the accept-reject algorithm (ARA) [70,71], is an effective and relatively simple to implement method for reducing the respiratory motion; however, the drawback is that the scan time is inversely proportional to the size of the gating window leading to an even further extended scan time for small gating windows. The magnitude of the diaphragmatic motion is normally around 15–30 mm, however a navigator gating window of 5 mm diaphragmatic motion is often used which leads to a navigator efficiency (the number of accepted navigator positions divided by the total number of navigator acquisitions) of approximately 25%–50%, occasionally resulting in a quadrupled scan time (or more) in the event of irregular breathing or respiratory drift. An alternative to using a fixed respiratory gating window with variable and unpredictable navigator efficiency is to predefine the navigator efficiency, which leads to a variable gating window size but predictable scan time [72].

A retrospective gating method was described by Li *et al.* [73]. The approach involves oversampling data with a certain factor, typically five, during free breathing. For each data point, out of the five acquired samples, the one with the least amount of respiratory motion is used for reconstruction. However, the total scan time becomes greatly increased, proportional to the oversampling factor, and the technique is sensitive to respiratory drift where possibly all five data acquisitions for a certain *k*-space point could be acquired with a large displacement relative to other data points. More efficient gating strategies have been proposed, typically involving some sort of ordering scheme whereby *k*-space data acquired in the central area of *k*-space is gated to a quiescent phase of the respiratory cycle, and vice-versa the outer *k*-space lines are acquired in a phase with more respiratory motion. As the central lines contain most of the image energy, motion during their acquisition is more likely to cause motion artifacts. Respiratory ordered phase encoding (ROPE) [74], centrally ordered phase encoding (COPE) [75] and hybrid ordered phase encoding (HOPE) [76] are three such methods which were implemented for 2D cardiac imaging. ROPE was extended to 3D and used for CMRA and shows improvements compared to the ARA [77].

The diminishing variance algorithm (DVA) is a gating technique which does not utilize a predefined gating window, but instead terminates when the acquired navigator variance reaches a certain thresh-hold [78]. First, a complete dataset is acquired allowing navigator positions throughout the whole respiratory cycle. In the following acquisitions the data which has been acquired with the largest navigator values (furthest away from end-expiration) is re-acquired, thus reducing the total variance of the navigator acquisition. This is repeated until the navigator variance reaches a predefined threshold or exceeds a time limit. Phase encoding with automatic window selection (PAWS) [79] is a gating approach which divides the respiratory cycle in to several bins and each bin is assigned a starting point in *k*-space. The scan is terminated when data from three adjacent bins are fully acquired. Recently, this approach has been extended to 3D radial phase encoding imaging which allows for a smoother motion distribution between bins [80]. The performance of retrospective gating, ARA, DVA and PAWS were compared in a study by Nguyen *et al.* [81] which found PAWS to be the most effective and efficient for free breathing CMRA.

## 4. Respiratory Motion Correction Strategies

Respiratory motion correction can be either prospective or retrospective, where prospective correction involves estimating the motion before every acquired *k*-space segment, using previously described motion estimation techniques, and correcting for it by updating the gradient, excitation and acquisition parameters accordingly. Retrospective correction involves correcting for the motion after the scan is completed, which relaxes the real-time constraints for motion estimation, but prohibits adjustment of slice position due to through-plane motion. It is worth mentioning that prospective motion correction is limited to affine corrections, a set of linear transformations including translation, rotation, scaling and shearing which can be applied for prospective motion correction by adapting the linear gradient waveforms [82], whereas retrospective motion correction can be used for correction with more degrees-of-freedom, such as non-linear correction [69]. Although respiratory motion estimation typically precedes motion correction, this is not a strict requirement. A method has been developed whereby the respiratory motion is iteratively corrected without prior motion estimation by maximizing an edge sharpness metric in image space [83].

### 4.1. Translational Correction

Translational correction, in conjunction with ARA gating, is the most commonly used respiratory motion compensation approach, as respiratory motion can be well approximated by translational motion in the end-expiratory phase. The translational motion *Δa_x_*, along encoding direction *x*, creates a phase shift *θ_x_* inversely proportional to the image field-of-view, *FOV_x_*, and can be calculated as:

(1) $$\theta_{x}=\frac{2\pi \cdot \Delta a_{x}}{{\text{FOV}}_{x}}$$

For prospective translational correction of in-plane motion, the spatial encoding gradients can be appropriately adjusted, and if translational correction is performed in the through-plane direction (slice encoding) then the phase of the RF pulse and A/D sampler will be modulated as well, resulting in a corresponding change in position of the excited imaging plane. For obliquely aligned, small slab CMRA such as targeted scans, through-plane respiratory motion may be substantial which requires prospective correction [84]. Whole-heart CMRA [85] with an axial orientation experiences the dominant respiratory motion component (FH motion) along the slice encoding direction which may introduce significant through-plane motion unless prospective correction is used. Nevertheless, the absolute respiratory induced displacement of the heart is typically on the order of magnitude smaller than the 3D FOV of whole-heart CMRA, particularly for coronal or sagittal orientations, which mitigates the effects of through plane motion and enables the use of retrospective translational correction. Typically, retrospective translational motion is performed in *k*-space, where the phase shift *θ_x_* can be applied to the raw data sample ***k****_j_* at *k*-space index *j* acquired along encoding direction *x* as:

(2) $${\text{k}}_{j}^{\prime }={\text{k}}_{j}\cdot e^{-i\cdot \theta_{x}\cdot j}$$

where ***k***′*_j_* is the motion corrected data.

### 4.2. Higher Order Correction

**Figure 6.**
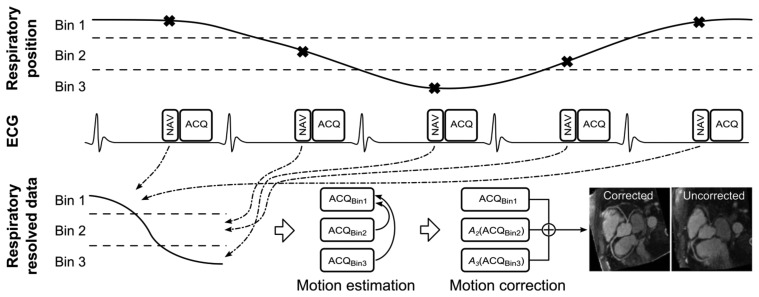
Schematic of a general respiratory binning, motion estimation and correction procedure. The respiratory position is measured using a navigator (NAV) for every data acquisition (ACQ), which can then be associated to a respiratory bin. As respiration is cyclical this allows multiple ACQs to be associated to a particular respiratory bin. Data from each bin can be reconstructed separately (ACQ_Bin1-3_) and is now respiratory resolved, although undersampled. For radial binning approaches the undersampled data for each bin can be used to estimate motion between bins [67,68]. However, for Cartesian binning approaches additional fully sampled data can be acquired [11,69]. The respiratory motion is typically estimated by registering ACQ_Bin2_ and ACQ_Bin3_ to the end-expiratory ACQ_Bin1_ to generate the transformations *A_2_* and *A_3_*. The motion corrected CMRA data can be obtained by applying these transformations to the CMRA data in the corresponding bins and summing the results. Note, in this example 3 respiratory bins are used, however in practice 4 to 6 bins are typically employed. Adapted from [67].

While translational correction is adequate in the majority of cases if the CMRA scan is gated to the most quiescent respiratory phase, rotation and non-rigid motion correction such as affine correction has to be considered if no respiratory gating is employed [7], which is desirable to minimize scan time. Affine models describe a combination of linear transformations including rotation, shear and scale, as well as translation. A 3D affine transformation *A* transforms the point r = [*x, y, z, 1*]*^T^* to r′ = [*x*′, *y*′*, z*′*, 1*]*^T^* according to:

(3) $$\left[\begin{array}{c} x^{\prime }\\ y^{\prime }\\ z^{\prime }\\ 1 \end{array}\right]=\left[\begin{array}{cccc} M_{00} & M_{01} & M_{02} & T_{x}\\ M_{10} & M_{11} & M_{12} & T_{y}\\ M_{20} & M_{21} & M_{22} & T_{z}\\ 0 & 0 & 0 & 1 \end{array}\right]\;\left[\begin{array}{c} x\\ y\\ z\\ 1 \end{array}\right]$$

where *t* = [*T_x_*, *T_y_*, *T_z_* ]*^T^* denotes the 3D translational components and the sub-matrix ***M*** = ***A***(4;4) defines the rotation, scale and shear. Prospective 3D affine correction has been implemented for CMRA in which the affine motion model is calculated from a calibration scan and a d1D NAV used to steer the model during the CMRA scan, which allows for data acquisition throughout almost the entire respiratory cycle [50]. Previously described approaches using respiratory binning have been used to retrospectively correct for affine and non-linear motion, either in image space [11,67] or in *k*-space [68,69], with reported navigator efficiencies approaching 100%. Affine correction in *k*-space can be performed based on the following relationship:

(4) $$F\prime (\text{k}\prime )=\frac{e^{i2\pi (\frac{{\text{k}}^{\text{T}}\text{t}}{{\text{M}}^{T}})}}{\left|\text{det}(\text{M})\right|}F({\text{M}}^{-T}\text{k})$$

where *F(****k****)* denotes the uncorrected and *F*′*(****k***′*)* the corrected *k*-space. Non-linear correction can be performed by generalizing the signal equation to also incorporate the different motion states [86]. In this way, the motion corrupted *k*-space *F(****k****)* can be written as:

(5) $$F(\text{k})=\sum_{s}{\text{W}}_{s}\;{\text{EP}}_{s}\text{o}$$

where ***o*** represents the motion-free imaged object, ***E*** the data encoding matrix, ***W****_s_* and ***P****_s_* the non-linear warping and data sampling operators, respectively, at motion state *s*. The motion corrected image can be obtained by performing matrix inversion on Equation (5). A generalized schematic of motion correction using respiratory binning is shown in Figure 6, where images reconstructed for different bins can be used to calculate and thereafter apply affine or non-linear transformations between each bin, to allow for 100% navigator efficiency.

A drawback of higher order motion correction is that the data post-processing becomes increasingly computationally expensive and time-consuming as respiratory motion estimation and correction is performed with more degrees of freedom. Excessive post-processing durations may impede the clinical utility of advanced motion correction strategies, although in many cases the post-processing can be expedited by the use of parallel computing [87].

## 5. Conclusions

The problem of respiratory motion has plagued the field of free-breathing CMRA for many years and yet it remains a largely unsolved challenge. In the last decade the predominant research trend has been moving away from diaphragmatic navigators with motion models of varying complexity and towards direct motion compensation of the respiratory induced heart motion. This includes self-navigation and image based-navigation techniques, the former offering improved ease-of-use while the latter provides motion correction with more degrees-of-freedom. Furthermore, improved motion estimation accuracy allows for advanced motion correction algorithms incorporating affine and non-linear correction, obviating the need for respiratory gating. Despite the potential of some of these advanced methods to compensate for almost all of the respiratory motion, it is unlikely that they will receive widespread clinical use unless they can be seamlessly integrated into the clinical workflow, and without adding complexity to the cardiovascular MRI examination. The main aim of motion compensation in CMRA should not only be to make the scans faster and more robust towards respiratory motion, but also make the scans easier to perform so as to reduce operator dependence and requirement for specialized training. So far, advanced compensation approaches have only been used in small research studies involving healthy volunteers whereas most patient studies have been limited to simple diaphragmatic motion compensation techniques. Further work is required to assess the clinical merits of these emerging approaches. To this end, a focus on integrating the new methods into clinical practice is needed, including maximizing ease of use and minimizing post-processing times.
